# 1621. "False-Positive" Cytomegalovirus (CMV) Screening Tests in Neonates: A Real Dilemma!

**DOI:** 10.1093/ofid/ofad500.1456

**Published:** 2023-11-27

**Authors:** Zachery S Lewald, Rachelle Crisan, Traci Pifer, Conception De Alba Romero, Masako Shimamura, Asuncion Mejias, Alexandra Katherine Medoro, Doug P Salamon, Huanyu Wang, Amy Leber, Ursula M Findlen, Oliver Adunka, M D S Malhotra, Pablo J Sanchez

**Affiliations:** Nationwide Children's Hospital, Delaware, Ohio; Nationwide Children's Hospital, Delaware, Ohio; Nationwide Children's Hospital, Delaware, Ohio; Nationwide Children's, Columbus, Ohio; Nationwide Children's Hospital, Delaware, Ohio; St Jude Children's Researh Hospital, Memphis, Tennessee; Nationwide Children's Hospital, Delaware, Ohio; Nationwide Children's Hospital, Delaware, Ohio; Nationwide Children's Hospital, Delaware, Ohio; Nationwide Childrens Hospital, Columbus, Ohio; Nationwide Children's Hospital, Delaware, Ohio; Nationwide Children's Hospital, Delaware, Ohio; Nationwide Children's Hospital - The Ohio State University, Columbus, OH; Nationwide Children's Hospital - The Ohio State University, Columbus, OH

## Abstract

**Background:**

PCR testing of clinical specimens has been associated with false-positive test results. Detection of CMV DNA by PCR in saliva and urine has been used to identify neonates with congenital CMV infection, but the frequency of false-positive PCR results is not fully known. Our objective is to describe the frequency of "false-positive" saliva and urine CMV PCR tests when performed as screening for congenital CMV infection.

**Methods:**

From 2018 to 2023, neonates were screened for CMV infection if they had abnormal clinical, laboratory, or radiographic findings, referred on the newborn hearing screen, and since 2019, on admission to the 9 neonatal intensive care units (NICU) of the Nationwide Children’s Hospital neonatal network, Columbus, OH. Pertinent demographic, clinical, audiologic, laboratory, and radiographic data were obtained and managed using REDCap. Infants who had an initial positive PCR screening test for CMV but subsequent negative PCR tests were identified.

**Results:**

During the 6 year study period, 139 infants were diagnosed with congenital CMV infection. An additional 28 (17%) neonates tested positive for CMV DNA in saliva (n=26) or urine (n=2) but subsequent tests of urine specimens were negative for CMV. Most of the infants were tested as part of universal NICU screening (n=22), 5 had symptomatic disease, 3 referred on the newborn hearing screen, and 1 had thrombocytopenia. The infants were tested at a median (IQR) age of 3 (2-6) days. Their median (IQR) birth weight and gestational age were 2458 (1865-3360) grams and 37 (33-39) weeks, respectively. Four infants had their dried blood spot tested for CMV by PCR and were negative. 17 (77%) of 22 infants had known maternal milk exposure in the neonatal period.

**Conclusion:**

Neonatal saliva and/or urine screening for congenital CMV infection was associated with a “false-positive” test result in 17% of neonates who tested positive for CMV DNA by PCR. These results suggest that confirmation of a diagnosis of congenital CMV infection by PCR requires testing of multiple specimens with one of them being urine.

**Disclosures:**

**Asuncion Mejias, MD, PhD, MsCS**, Astra-Zeneca: Advisor/Consultant|Merck: Grant/Research Support|Pfizer: Advisor/Consultant|Sanofi-Pasteur: Advisor/Consultant **Alexandra Katherine Medoro, MD**, Merck: Grant/Research Support **Amy Leber, PhD**, Biomeriux: Grant/Research Support|BioRad: Advisor/Consultant|Cephied: Grant/Research Support|Diasorin: Grant/Research Support

